# In Vitro Analysis of Wearing of Hip Joint Prostheses Composed of Different Contact Materials

**DOI:** 10.3390/ma14143805

**Published:** 2021-07-07

**Authors:** Jian Su, Jian-Jun Wang, Shi-Tong Yan, Min Zhang, Hui-Zhi Wang, Ning-Ze Zhang, Yi-Chao Luan, Cheng-Kung Cheng

**Affiliations:** 1 School of Biological Science and Medical Engineering, Beihang University, Beijing 100083, China; su8318@163.com (J.S.); nzzhang@buaa.edu.cn (N.-Z.Z.); qluanyichao@163.com (Y.-C.L.); 2 Beijing Institute of Medical Device Testing, Beijing 101111, China; wangjianjun@bimt.org.cn (J.-J.W.); yanshitong@bimt.org.cn (S.-T.Y.); 3 Beijing Advanced Innovation Center for Biomedical Engineering, Beihang University, Beijing 100083, China; m.zhang@buaa.edu.cn; 4 School of Biomedical Engineering, Shanghai Jiao Tong University, Shanghai 200240, China; wang_huizhi8866@163.com

**Keywords:** hip prosthesis, acetabular liner, femoral head, wear rate, XLPE

## Abstract

Cobalt-chromium-molybdenum alloy (CoCrMo) and ceramic are the two most common materials for the femoral head in hip joint prostheses, and the acetabular liner is typically made from ultra-high molecular weight polyethylene (UHMWPE), highly cross-linked polyethylene (XLPE), or highly cross-linked polyethylene blended with Vitamin E (VEXLPE). The selection of suitable materials should consider both wear performance and cost-effectiveness. This study compared the wear rate between different friction pairs using a hip joint simulator and then recommended a suitable prosthesis based on the corresponding processing technology and cost. All wear simulations were performed in accordance with ISO 14242, using the same hip joint simulator and same test conditions. This study found that when using the same material for the femoral head, the XLPE and VEXLPE liners had a lower wear rate than the UHMWPE liners, and the wear rate of the XLPE liners increased after blending with Vitamin E (VEXLPE). There was no significant difference in the wear rate of XLPE when using a CoCrMo or ceramic head. Considering the wear rate and cost-effectiveness, a CoCrMo femoral head with an accompanying XLPE liner is recommended as the more suitable combination for hip prostheses.

## 1. Introduction

Osteoarthritis (OA) is one of the most common diseases of the hip joint. It is clinically characterized by joint pain, deformation, and restricted movement [1]. Late-stage OA often requires surgical intervention, such as total hip arthroplasty (THA). However, postoperative complications after THA are common and can result in patient disability, implant failure, osteolysis, and prosthesis loosening [2]. It has been reported that over 50% of THA revisions are the result of implant loosening [3]. Wear particles generated between the femoral head and acetabular liner as the joint articulates are engulfed by macrophages to produce a large number of cytokines which activate osteoclasts and can cause osteolysis around the prosthesis, subsequently leading to prosthesis loosening [4,5]. Therefore, the wear performance of the hip joint prostheses is critical for long-term implant survival [6,7].

At present, the most widely used materials for the femoral head are cobalt-chromium-molybdenum alloy (CoCrMo) and ceramic, and the acetabular liner is typically made from ultra-high molecular weight polyethylene (UHMWPE), highly cross-linked polyethylene (XLPE), or highly cross-linked polyethylene blended with Vitamin E (VEXLPE) [8]. Studies have shown that XLPE liners have a lower wear rate than UHMWPE [9,10] and that the addition of vitamin E to XLPE can further improve the anti-aging performance and wear resistance over UHMWPE liners [11,12]. It has also been reported that ceramic femoral heads offer superior wear resistance to CoCrMo heads [13].

Due to the large number of cycles required, the time-consuming nature of abrasion tests, and their associated costs, most studies to date conducted a comparative analysis of only one or two friction pairs. For example, Lizeth Herrera et al. only evaluated the wear performance of XLPE and UHMWPE liners [9], while Vesa Saikko assessed the impact of adding Vitamin E to XLPE [11]. Due to differences in test equipment, the methods, and the conditions simulated, there can be considerable variation in the results retrieved. Lizeth Herrera et al. reported an average wear rate of 1.35 ± 0.68 mm^3^/mc after 5 million cycles using MTS equipment and calf serum diluted to 20 g/L [9], whereas Hermida et al. reported a wear rate of 16.92 mg/mc using AMTI equipment and 90% calf serum [14]. Using AMTI equipment and a lubricant of undiluted bovine serum, Estok et al. performed abrasion tests on irradiated XLPE and recorded wear rates of −0.42 mg/mc for a 9.5-Mard gamma irradiated material and 1.2 mg/mc for a 5-Mard material [15]. In addition to the inconsistent results reported, these studies only considered a small selection of materials commonly used in acetabular liners. Few studies have evaluated the wear performance of different combinations of head and liner materials.

The purpose of this study is to evaluate how different combinations of materials for the femoral head and acetabular liner affect the wear rate of artificial hip joint prostheses. This study hypothesized that a ceramic head paired with a VEXLPE liner would have the lowest wear rate.

## 2. Materials and Methods

This study consisted of 5 groups with different contact materials: ceramic head and XLPE liner, ceramic head and VEXLPE liner, CoCrMo head and XLPE liner, and CoCrMo head and UHMWPE liner. With reference to ISO 14242-1:2014 [16], ISO 14242-2:2016 [17], five samples were used for the wear test of each group of contact materials (combination of a femoral head and acetabular liner). Three of the five samples in each group were randomly chosen as tested samples and the remaining two (or one) were control samples for the calculation of wear loss. The head and liner combinations chosen were commercially available total hip prostheses (Table 1). The materials used to manufacture the liners were produced by the same company (Quadrant, Switzerland) using the same manufacturing processes, such as polyethylene resins, crosslinking dose or crosslink density, post-irradiation thermal treatments, and incorporation methods. The unformed materials were then processed into liners by the Manufacturers stated in Table 1. Except for sizing, the ceramic heads were identical. All products conformed to YY 0118-2016 “Joint replacement implants—Hip joint prostheses” [18] (equivalent to the ISO 7206-2 “Implants for surgery—Partial and total hip joint prostheses—Part 2: Articulating surfaces made of metallic, ceramic, and plastics materials” [19]). The liners in this study had a surface roughness of 0.3–0.5 μm, a thickness of 5–7 mm, and a roundness of 50–70 μm. The femoral heads in this study had a surface roughness of 0.010–0.015 μm, and a roundness of 2–5 μm.

### 2.1. Samples

Different sizes of ceramic and CoCrMo femoral heads were paired with XLPE, VEXLPE, and UHMWPE liners to create 5 groups (Table 1). The wear rates were compared under the same test conditions developed in accordance with ISO 14242-1:2014 [16], ISO 14242-2:2016 [17].

### 2.2. Wear Tests

**Figure 1:**
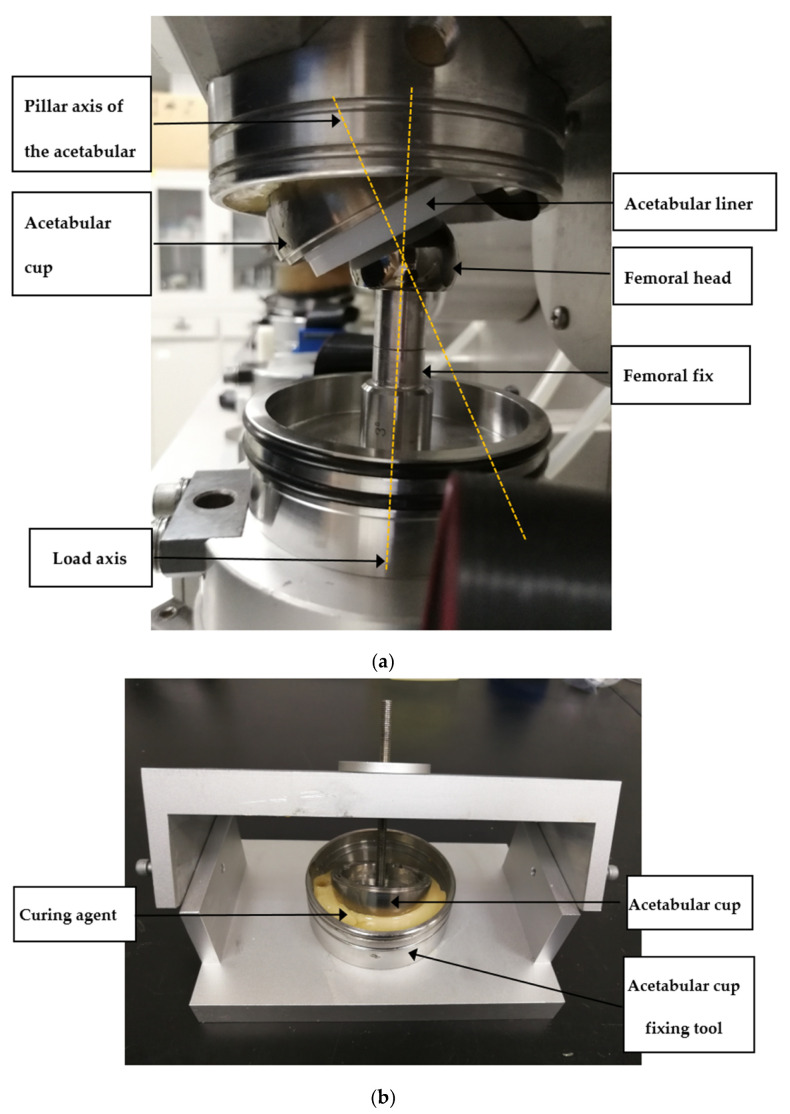
(**a**) Fixation in the hip joint simulator. (**b**) Fixation of the acetabular cup.

An AMTI joint prosthesis simulator (ADL-H12-01, AMTI, Watertown, MA, USA) (Figure 1) was used for all wear tests, which were performed in accordance with ISO 14242-1:2014 [16], as detailed below.

All samples were weighed on a high precision analytical balance (XS205DU, Mettler Toledo LLC, Columbus, OH, USA) with a maximum weight capacity of 220 g and a resolution of 0.01 mg.

The liners were first pre-soaked in calf serum for 2 days. The liners were then repeatedly washed, dried, and weighed until the mass change of each sample exceeded 24 h after being less than 10% of the cumulative mass change of the sample, as specified in ISO 14242-2:2016 [17].

After a constant weight was achieved, the femoral head was mounted in a custom rig. The focus of this study is the wear performance of the contact materials, and so only the wear produced by the articulating surfaces was considered. Therefore, the acetabular liner and the acetabular cup were directly secured in a rig (acetabular cup fixing tool) using a curing agent, and the rig was connected to an actuator on the simulator, which could simulate abduction, adduction, flexion, and extension of the hip joint (Figure 1a). As specified in ISO 14242-1:2014 [16], the acetabular component was tilted 30° ± 3° in the axial direction (Figure 1b). All three test specimens were subject to flexion/extension, abduction/adduction, inward/outward rotation, and an axial force using the input values recommended in ISO 14242-1:2014 [17]. Variations in the angular movement of the femoral component relative to the acetabular component were −18° to +25° for flexion/extension, −10° to +2° for inward/outward rotation, and −4° to +7° for abduction/adduction. The axial force ranged between 300 and 3000 N in one loading cycle.

Calf serum was diluted with deionized water to obtain a solution with a protein content of 30 g/L to simulate human synovial fluid. After every 500,000 cycles, the serum was completely replaced, and each test specimen was moved to a different simulator to minimize the impact of system errors.

The temperature of the serum was controlled at 37 °C using an antiseptic agent consisting of 0.2% NaN3 and 5 mmol/L EDTA.

5 million cycles of wear test were performed for each group.

The gravimetric wear and wear rate were calculated according to ISO 14242-2:2016 [17]. The gravimetric wear ($W_{n}$) referred to the net loss of mass from each test specimen after *n* loading cycles and was calculated using Equation (1). The gravimetric wear of all the test specimens was calculated at each testing stage during the wear simulation (500,000, 1 million, 2 million, 3 million, 4 million, and 5 million cycles).

(1) $$\begin{array}{c} W_{n}=m_{0}-m_{n}+S_{n}\\ S_{n}=\bar{m_{n}}-\bar{m_{0}} \end{array}$$

$m_{0}$—the mass of the test specimen before the wear test
$m_{n}$—the mass of the test specimen after *n* loading cycles
$S_{n}$—the increase in mass of the control specimen over the same period
$\bar{m_{0}}$—the mass of the control specimen before the wear test
$\bar{m_{n}}$—the mass of the control specimen after *n* loading cycles

The wear rate ($a_{G}$) was calculated as the least-squares linear fit relationship between $W_{n}$ and the number of loading cycles (*n*) using Equation (2).

(2) $$Wn=a_{G}\times n+b$$

*Wn*—the net loss in quality of the test specimen after *n* load cycles
*n*—cycles
*b*—constant

The average wear rate for the three test specimens was taken as the wear rate for that group. The results of Group 1 and Group 2 were compared to understand how adding Vitamin E to XLPE affected the wear rate of the liner. Group 3 and Group 4 were analyzed to determine the relationship between the wear rate of the liner and the accompanying femoral head material. And the wear rate from Group 4 and Group 5 was compared to determine which material (XLPE and UHMWPE) offered superior wear resistance when using a femoral head of the same size and material.

## 3. Results

**Figure 2:**
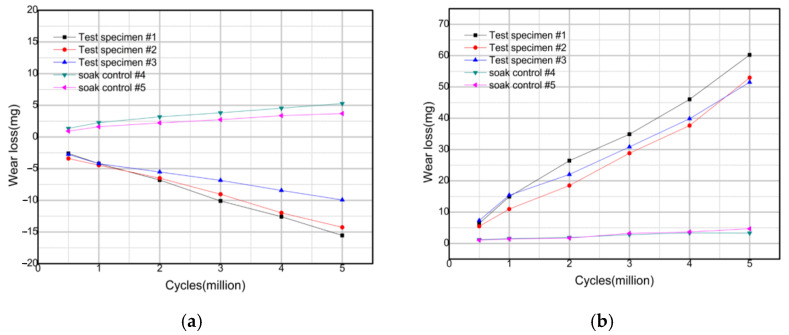

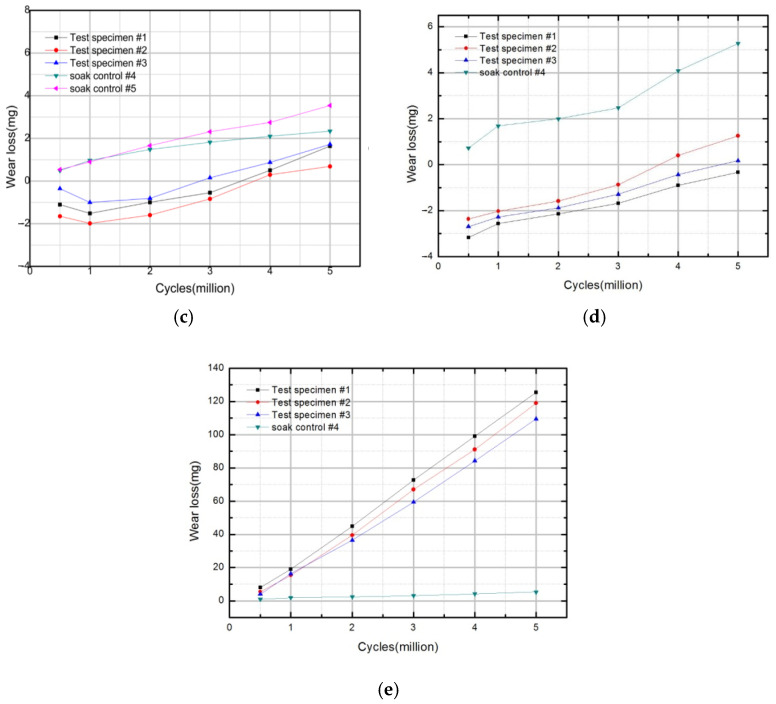
Gravimetric wear of the five test groups: (**a**) Group1; (**b**) Group2; (**c**) Group3; (**d**) Group4; (**e**) Group5 (see Table 2).

The gravimetric wear of each test sample during the wear test is given in Figure 2. The wear rate of the five groups is shown in Table 2.

### 3.1. Ceramic Head with XLPE and VEXLPE Liners

When combined with a 36 mm ceramic head, the wear rate of the XLPE liner with Vitamin E (VEXLPE, Group 2) had a considerably higher wear rate (10.45 ± 1.01 mg/million cycles) than the plain XLPE liner (Group 1) (−2.48 ± 0.64 mg/million cycles). The addition of Vitamin E had a detrimental effect on the wearing of the liner. The negative wearing may be explained by the acetabular liner absorbing calf serum during the wear process. While each of the liners will absorb a certain amount of calf serum, when the amount of serum absorbed by the acetabular liner exceeds the loss in material mass from the liner, such as with XLPE (Group 1), negative wear will appear.

### 3.2. Ceramic and CoCrMo Femoral Heads with XLPE Liner

Ceramic and CoCrMo femoral heads of diameter 28 mm were assembled with an XLPE acetabular liner and assigned to Groups 3 and 4, respectively. The wear rate from these two groups was very similar, with rates of 0.60 ± 0.21 mg/million cycles and 0.68 ± 0.11 mg/million cycles respectively.

### 3.3. CoCrMo Femoral Head with XLPE and UHMWPE Liners

A 28 mm CoCrMo femoral head was paired with an XLPE liner (Group 4) and UHMWPE liner (Group 5). The results showed that the XLPE liner had a much lower mean wear rate (0.68 ± 0.11 mg/million cycles) than the UHMWPE liner (24.97 ± 1.59 mg/million cycles).

## 4. Discussion

The wear mechanisms of friction components depend on the conditions of articulation, material properties, and the surface topography of the co-acting parts [20]. The wear mechanisms of the polymeric socket resulted from several phenomena; including plastic deformation, abrasive wear, fatigue, and adhesion [20,21]. Therefore, it is important to examine these determinants to improve the durability of a friction pair.

The conditions of articulation of the samples selected in this study are kept the same, and the surface topography (surface roughness, roundness) of the friction joint articular surface is also kept within the manufacturing tolerance range, so the material is hypothesized to be the main factor affecting the wear performance in this study.

**Figure 3:**
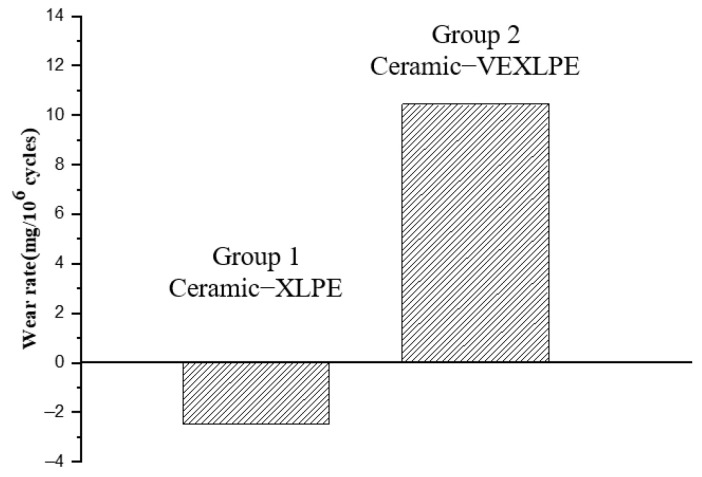
Wear rate for Group 1 and Group 2.

This study found that the XLPE and VEXLPE liners had a lower wear rate than the UHMWPE liners, and the wear rate of the XLPE liners increased after adding Vitamin E (Figure 3). However, changing the material of the femoral head had little effect on the wear rate.

It is known that the volume and rate of fluid uptake are affected by whether the bearing is in motion [15]. Therefore, the mass change of the absorbed serum by the control sample (only subjected to axial load) in each group does not accurately represent the amount of serum absorbed by the test sample (simulating human gait movement). Moreover, after the prosthesis absorbs serum, it is difficult to completely remove the serum through dehydration and deproteinization using the cleaning process described in ISO 14242-2:2016 [17].

Affatato et al. reported a wear rate of 16.1 ± 8.2 mg/million cycles for a VEXLPE liner combined with a CoCrMo femoral head [22]. In this current study, Group 4 combined an XLPE liner with a CoCrMo head but recorded a considerably lower wear rate of 0.68 ± 0.11 mg/million cycles. Although these studies used a different-sized femoral head, the results clearly show that adding Vitamin E to the liner can drastically increase the wear rate. The reason may be that the addition of VE inhibits the amount of cross-linking of XLPE [21,23], resulting in a loss of wear resistance. Meneghini et al. [24] recorded the friction torque on ceramic and CoCrMo heads when paired with XLPE liners with and without Vitamin E. With the addition of VE, the torque on the ceramic head increased by 58% and increased by 31% on the CoCrMo head. The higher torque produces greater friction, which can increase the degree of wear. This may be one of the reasons why the wear resistance of XLPE was reduced after adding VE.

**Figure 4:**
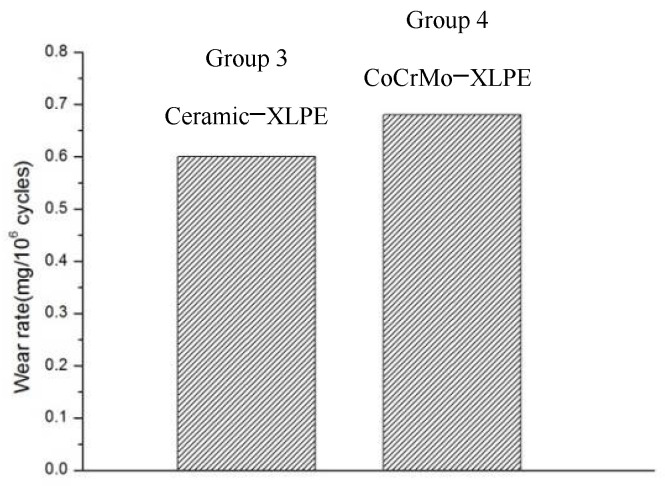
Wear rate for Group 3 and Group 4.

As can be seen in Figure 4, changing the head material has little effect on the wear rate of the liner. Similarly, Merola et al. [21] reported a wear rate of 4.09 ± 0.64 mm^3^/10^6^ cycles for a CoCrMo-XLPE combination and 3.35 ± 0.29 mm^3^/10^6^ cycles for an Alumina-XLPE combination. Therefore, compared with a UHMWPE liner, XLPE offers better wear resistance [10].

**Figure 5:**
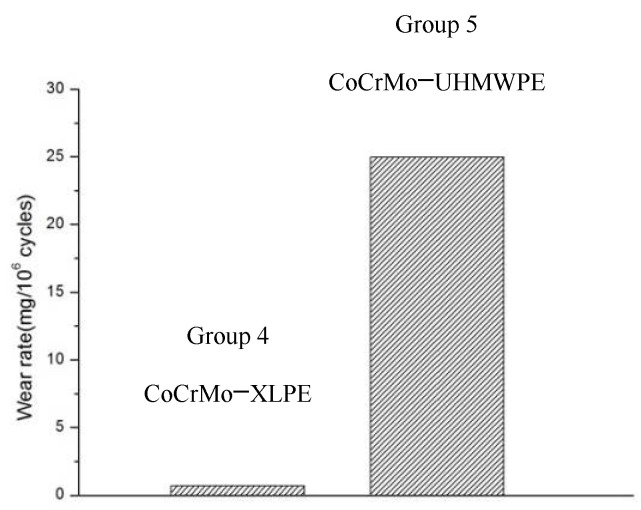
Wear rate for Group 4 and Group 5.

Figure 5 clearly shows a much lower wear rate for the XLPE liner than the UHMWPE liner. When paired with a 32 mm CoCrMo head, Lizeth et al. [9] reported a wear rate of 1.35 ± 0.68 mm^3^/10^6^ cycles for an XLPE liner and 46 ± 8.7 mm^3^/10^6^ cycles for UHMWPE, similar to previous findings reported in clinical follow-up studies [25]. Tipper et al. [13] reported a wear rate of 31.0 ± 4.0 mm^3^/10^6^ cycles (~29 mg/million cycles) for a UHMWPE liner paired with a 28 mm ceramic head, which is much greater than the wear rate observed in Group 3 of 0.60 ± 0.21 mg/million cycles. In conclusion, XLPE liners have a much lower wear rate than both VEXLPE and UHMWPE liners irrespective of the material used for the femoral head.

The cost of materials is also an important consideration when selecting a suitable prosthesis; In China, UHMWPE liners range from ¥2000 (approximately $310)~¥5000 (approximately $770), XLPE liners from ¥5000 (approximately $770)~¥10,000 (approximately $1550), CoCrMo heads from ¥3000 (approximately $460)~¥6000 (approximately $920), and ceramic heads from ¥10,000(approximately $1550)~¥20,000 (approximately $3100) each.

There are some limitations to this study that should be noted. Firstly, because of the long cycle of the wear test and high cost, representative sizes were chosen for this study. The aim of this study was to evaluate implant wear from different material combinations, therefore all comparisons were carried out using similarly sized implants. Secondly, the amount of serum absorbed by the control group was not necessarily the same as the test group. However, each group used the same method to set the control group and the test group, according to the methods detailed in ISO 14242, which also allowed comparison between groups. Thirdly, as with all in vitro research, the methods used in this study cannot fully replicate the in vivo conditions of the hip joint. However, the aim of this study was to compare the wear performance of different materials, and so the methods used are adequate for this purpose.

## 5. Conclusions

This study found that the wear rate of XLPE liners was lower than both VEXLPE and UHMWPE liners. Also, importantly, the wear rate of XLPE increased after adding Vitamin E (VEXLPE). The use of a CoCrMo or ceramic head had little effect on the wear resistance of the XLPE liners. Because ceramic femoral heads are more expensive and the production process is more complicated, and considering the superior wear resistance of XLPE, a CoCrMo femoral head with an accompanying XLPE liner is potentially superior to other contact materials in terms of wear resistance and cost-effectiveness.

## Figures and Tables

**Table 1:** Prosthesis combinations used in this study.

Group Number	Material	Head Diameter (mm)	Manufacturer
1	Ceramic head & XLPE liner	36	Company A, Beijing Economic and Technological Development Zone, Beijing
2	Ceramic head & VEXLPE liner	36	Company B, Shunyi, Beijing
3	Ceramic head & XLPE liner	28	Company C, Haidian, Beijing
4	CoCrMo head & XLPE liner	28	Company D, Haidian, Beijing
5	CoCrMo head & UHMWPE liner	28	Company E, Tuttlingen, Germany

**Table 2:** Wear rate after 5 × 10^6^ cycles.

Group Number	Head Diameter (mm)	Materials	Wear Rate of Liner (mg/million Cycles)	Mean Wear Rate (mg/million Cycles)
1	36	Ceramic−XLPE	−3.000	−2.48 ± 0.64
			−2.660	
			−1.770	
2	36	Ceramic−VEXLPE	11.600	10.45 ± 1.01
			10.070	
			9.690	
3	28	Ceramic−XLPE	0.640	0.60 ± 0.21
			0.610	
			0.540	
4	28	CoCrMo− XLPE	0.599	0.68 ± 0.11
			0.808	
			0.630	
5	28	CoCrMo−UHMWPE	26.333	24.97 ± 1.59
			25.362	
			23.223	

## Data Availability

The data is available from the corresponding author.
